# Trajectories of body mass index among Canadian seniors and associated mortality risk

**DOI:** 10.1186/s12889-017-4917-0

**Published:** 2017-12-04

**Authors:** Meng Wang, Yanqing Yi, Barbara Roebothan, Jennifer Colbourne, Victor Maddalena, Guang Sun, Peizhong Peter Wang

**Affiliations:** 1 Division of Community Health & Humanities, 300 Prince Philip Drive, St. John’s, NL A1B 3V6 Canada; 2 Discipline of Medicine, Faculty of Medicine, Memorial University, St. John’s, NF Canada

**Keywords:** Body mass trajectories, Seniors, Latent class growth modelling, Mortality

## Abstract

**Background:**

This study aims to characterize the heterogeneity in BMI trajectories and evaluate how different BMI trajectories predict mortality risk in Canadian seniors.

**Methods:**

Data came from the Canadian National Population Health Survey (NPHS, 1994–2011) and 1480 individuals aged 65–79 years with at least four BMI records were included in this study. Group-based trajectory model was used to identify distinct subgroups of longitudinal trajectories of BMI measured over 19 years for men and women. Cox proportional hazards models were used to examine the association between BMI trajectories and mortality risks.

**Results:**

Distinct trajectory patterns were found for men and women: ‘Normal Weight-Down’(N-D), ‘Overweight-Normal weight’ (OV-N), ‘Obese I-Down’ (OB I-D), and ‘Obese II- Down’ (OB II-D) for women; and ‘Normal Weight-Down’ (N-D), ‘Overweight-Normal weight’ (OV-N), ‘Overweight-Stable’ (OV-S), and ‘Obese-Stable’ (OB-S) for men. Comparing with OV-N, men in the OV-S group had the lowest mortality risk followed by the N-D (HR = 1.66) and OB-S (HR = 1.98) groups, after adjusting for covariates. Compared with OV-N, women in the OB II-D group with three or more chronic health conditions had higher mortality risk (HR = 1.61); however, women in OB II-D had lower risk (HR = 0.56) if they had less than three conditions.

**Conclusion:**

The course of BMI over time in Canadian seniors appears to follow one of four different patterns depending on gender. The findings suggest that men who were overweight at age 65 and lost weight over time had the lowest mortality risk. Interestingly, obese women with decreasing BMI have different mortality risks, depending on their chronic health conditions. The findings provide new insights concerning the associations between BMI and mortality risk.

## Background

The Canadian population is aging and the prevalence of obesity among the elderly is rising [1]. Obesity-related medical care costs associated with the elderly population are substantial [2]. Although there is clear evidence that excess body weight is associated with an increased risk of mortality in young to middle-aged adults [3, 4], the associations may not be the same for seniors.

Previous studies have found conflicting evidence on the associations between BMI and mortality in the elderly population [5]. Some studies reported J or U-shaped associations between mortality and BMI [6, 7], while others reported a positive linear relationship [4, 8]. These controversial findings are likely a result of the limited BMI measurements that were measured at one time point [9–12]. Consequently, these studies failed to detect the development of BMI and the impact of these BMI changes on longevity. It is documented that changes in BMI over time, rather than static BMI status, are more strongly predictive of mortality risk [13–15]. However, the majority of previous findings are based on modeling one average BMI pattern for the underlying population [16–20]. Latent Class Growth Modelling (LCGM) can identify different BMI trajectories within a population based on maximum likelihood methods [21, 22].

An important reason to identify distinct BMI trajectories is to investigate whether these trajectories carry differential health risks and mortality potentials [23]. Wang et al. found that subjects who were overweight or obese throughout their adulthood were more likely to have numerous health conditions compared with their healthy weight counterparts [24]. Zheng et al. used LCGM and reported that individuals in the overweight-stable group lived longer than individuals in the normal weight and obesity trajectory groups [13]. However, the covariates that were adjusted in the survival analyses were from the baseline interview only. Previous research has demonstrated that there are gender differences in the associations between mortality and BMI trajectory [23, 25], being physically active is inversely associated with mortality risk across all BMI levels [26], and smoking is independently associated with a higher risk of mortality in the general population [27]. Therefore, this study aims to identify BMI trajectories for subjects aged 65–79 years over 18 years (1994–2011) and to examine the mortality risk associated with these BMI trajectories among Canadian women and men. This will provide the evidence on effect of long-term obesity on mortality in senior Canadians.

## Methods

The Household component of the Canadian National Population of Health Survey (NPHS) was used in this study. It is a nationally representative longitudinal health survey conducted by Statistics Canada, addressing economic, social, demographic, occupational, and environmental correlates of health. The NPHS had a multi-stage complex design and followed 17,276 respondents of all ages from 1994/1995 to 2010/2011 biennially. Detailed information on the NPHS can be found elsewhere [28]. The study population included people aged 65–79 at baseline, 30% (weighted percent) of subjects were excluded because they had less than four BMI records out of nine measurements; the final analysis was limited to 1480 individuals. Although excluded participants were different from those included ones in terms of the distribution of sex, lifestyle factors (i.e. physically active, drinking, and smoking), long-term disability, and death (data available upon request), they were excluded in this study primarily because of the non-response to the NPHS that results in less than four BMI records.

### Basic trajectory variable and accelerated longitudinal design

BMI was used as the trajectory variable. The NPHS provides up to nine measures of BMI over a span of 18 years. BMI was derived in the NPHS by calculating weight in kilograms divided by the square of height in meters, except for pregnant women. Height and weight were self-reported in the NPHS.

This study examined BMI trajectories for those aged 65–94 based on individuals who were 65–79 years at baseline based on an Accelerated Longitudinal Design (ALD) [29]. An ALD can study age-related developmental trajectories over an extensive age span in a relative short follow-up period of study by pulling data from different overlapping age cohorts [29]. In a single cohort design, one age cohort is sampled at baseline and followed for a period of time, whereas the NPHS have various age cohorts and multiple single age cohorts were sampled and followed within an ALD for 18 years. Each cohort begins with a set age and is finished with another set age at a different time [29, 30]. By design, ALD collects “each individuals’ measurements which covers only part of the age range being studied”, thus each individuals’ measurements contribute to only part of the whole BMI growth curve [29]. The advantages of ALD include a shorter observational period than a single cohort design; also it would be less affected by attrition [31]. The trajectory variable was BMIs or imputed BMI in case of missing values, which was calculated and provided by the NPHS [28].

### Survival time

The survival time was defined as the number of months from the first interview until death or study ended.

### Statistical analysis

LCGM was used to identify the BMI trajectories and the other four latent variables. LCGM can deal with three types of data: continuous outcome (specified as censored normal distribution), binary outcome (specified as binary logit distribution), and count outcome (specified as Poisson distribution). Thus, five latent variables related to BMI, physical activity, smoking, drinking, and number of chronic condition were modeled using LCGM with appropriate distribution assumptions. Measures of goodness-of-fit of the LCGM were based on Bayesian Information Criterion, group membership (no less than 5%), and average posterior group membership probabilities (no less than 70%). Specifically, model selection started with one quadratic group, and more groups were added only if a better fit was detected using the above criteria. In addition, only the polynomial terms (quartic, cubic, or quadratic) with significant coefficients were retained before adding additional groups, but the linear terms were kept whether they were significant or not.

Cox proportional hazards models were used to examine if different BMI trajectories carry different mortality risks. The impact of BMI trajectories on survival time was considered as the main effect in this study. The covariates used in this study include age, race/ethnicity (white or non-white), educational attainment (defined as graduation from high school), place of residence (rural or urban), and presence of disability (yes or no), as well as latent variables: longitudinally being physically active, smoking, drinking, and development of number of chronic health conditions. The NPHS collected the information on the participation of the 20 activities (e.g., walking, swimming, gardening, cycling, etc.) during leisure time in the past 3 months. Energy expenditure was calculated by multiplying the frequency of the activity by the average duration of that activity by the relevant energy cost (kilocalories per kilogram of body weight per hour) of that activity. Respondents were classified as active if their average daily energy expenditure was more than 2.9 kcal/kg, as moderately active if their average daily energy expenditure was 1.5–2.9 kcal/kg, and as inactive if their average daily energy expenditure was less than 1.5 kcal/kg. In this study, we combined moderately active with active to capture the probability of being physically active trajectories. The data on smoking (current smoker vs the rest) and drinking (regular drinker vs the rest) habits were self-reported at each wave of the NPHS. Using LCGM of the longitudinal data for physical activity, smoking, and drinking, and the number of chronic conditions, we identified two distinct trajectory groups with different longitudinal patterns for those variables.

Three Cox proportional hazards models were fitted: unadjusted model, partly adjusted model (adjusting for age, race/ethnicity, and education), and fully adjusted model (further adjusting for presence of disabilities, place of residence, and the other latent variables). Furthermore, potentially meaningful interactions (i.e. age and the other covariates, BMI trajectory groups and the covariates) were tested. Statistical significant variables defined as *P* < 0.05 were retained in the model. In addition, proportional hazards assumption were checked for each variable in the final model. All analyses were weighted using the survey sampling weights provided by Statistics Canada to account for the multi-stage sampling scheme and represent the underlying general Canadian population. All analyses in this study were stratified by gender using SAS version 9.3 (SAS Institute).

### Ethical considerations

This research was approved by Statistics Canada.

## Results

Of the 1480 individuals (aged 65–79) included in this study, 62.2% were women and 37.8% were men. They were predominantly white (95.7%), and 49.3% of the individuals reported having a high school education or higher at baseline. From 1994 to 2011, the prevalence of overweight (25 ≤ BMI < 30 kg/m2), obese (BMI ≥ 30 kg/m2) slightly decreased from 43.6% to 37.2%, and 14.6% to 9.3%, respectively. On the other hand, the weighted percent of underweight (BMI < 18.5 kg/m2) and normal weight (18.5 ≤ BMI < 25 kg/m2) increased from 1.7 to 4.5% and 40.1 to 50.0%, respectively. In addition, 79.3% of respondents reported having more than one chronic condition, 22.7% reported having long-term disabilities, and 46.5% rated their health as “very good” or “excellent.” Further, 54.1% of the 560 men and 36.9% of the 920 women in this study died over the duration of follow-up (18 years).

**Fig. 1:**
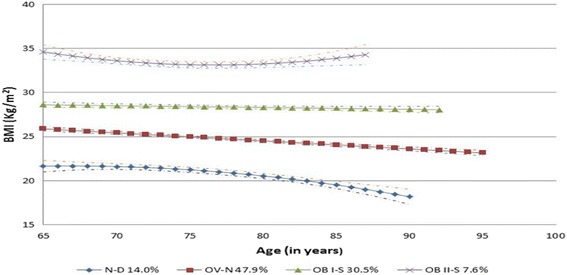
BMI trajectories for men (65–96 years), with 95% confidence intervals (four group model, no covariates included), NPHS, 1994–2011

**Fig. 2:**
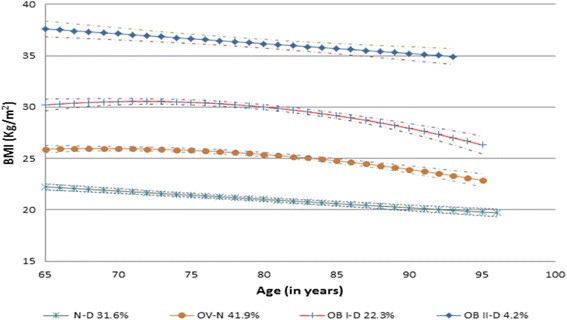
BMI trajectories for women (65–96 years), with 95% confidence intervals (four group model, no covariates included), NPHS, 1994–2011

LCGM identified four BMI trajectory groups and two trajectory groups for the other latent variables. Figure 1 reveals ‘Normal Weight-Down’ (N-D), ‘Overweight-Normal weight’ (OV-N), ‘Overweight-Stable’ (OV-S), and ‘Obese-Stable’ (OB-S) for men. Figure 2 shows ‘Normal Weight-Down’ (N-D), ‘Overweight-Normal weight’ (OV-N), ‘Obese I-Down’ (OB I-D), and ‘Obese II- Down’ (OB II-D) for women. The average posterior probability value of each trajectory group exceeded 0.90, which shows excellent model fit [21]. LCGM identified two trajectories for the probability of being physically active: close to 70% of subjects had low probabilities (*p* = 0.25) of being active at age 65 and this probability was decreasing over time; on the other hand, about 30% of seniors had high probabilities (*p* = 0.82) of being active at age 65 and this probability was also decreasing over time (Appendix Figure A1). As shown in Appendix Figure A2, 89% of subject have very low probabilities (*p* < 0.1) of smoking and this didn’t change significantly over time; the rest of sample had high probability (*p* = 0.95) of smoking while this probability was decreased greatly over time, the probability of smoking for this group was less than 0.3 when seniors reach their 90s. In addition, LCGM identified two trajectories for the development of the number of chronic conditions: less than three chronic conditions (57%), more than three chronic conditions (43%). Specifically, about 57% of the subjects had less than one chronic conditions and this number was increasing to an average of 3 at the age of 96. The other group started with more than two chronic conditions then also had more health conditions over time with an average of 5 at the age of 96.

Figure 1 shows that the group membership probability (GMP) of the N-D group in men was 14.0%, it started with a normal weight status at age 65 years, with an average BMI of 21.7, and slowly declined to an average BMI of 16.2 at the age of 96 years. It is likely that few men in the N-D group reported their BMI or were still alive after 90 years of age. Most individuals in this group remained underweight or normal weight between the ages of 65 and 96 years. The GMP of the OV-N group was 47.9%, and started with an overweight status at age 65, but gradually decreased to a normal weight status around 70–80 years of age then continued to decrease with increasing age, but did not reach underweight by age 96. The GMP of the OV-S group was 30.4% in men. The OV-S group started with an overweight status at age 65 years, with an average BMI of 28.0, and declined to an average BMI of 25.9 at age 96 years. The GMP of the OB-S group was 7.6% in men. This group started with an obese class I status at the age of 65 years, with an average BMI of 34.6, and remained in the obese class I range until the age of 90 years. It is likely that few men in the OB-S group reported their BMI or were still alive after 90 years of age.

Table 1 demonstrates that 50.4%, 69.7%, 48.9%, and 69.2% of men died in the OV-N, N-D, OV-S, and OB-S groups among men, respectively. Comparing with OV-N, the N-D group and the OB-S group were significantly associated with an 86% (*P* < 0.001) and 97% (*P* < 0.05) increase in mortality risk in the unadjusted model. Adjustment for age, race/ethnicity, and education had a minor effect on the HRs for the associations between the BMI trajectories and mortality risks. Specifically, the mortality risk associated with the N-D and OB-S groups slightly increased to 94% (P < 0.001) and 101% (P < 0.05). The increased risk was 66% (*P* = 0.003) for the N-D group and was 98% (P = 0.003) for OB-S in the fully adjusted model, when comparing with the OV-N group. The OV-S group was not significantly different in mortality risk from the OV-N group in any of the three models.

**Table 1:** Adjusted hazard ratios of BMI trajectories among men from cox proportional hazard models in the NPHS,1994–2011

	Men						
	Unadjusted model			Adjusted for demographics Factors^a^		Fully adjusted model^b^	
	N(%)death	HR(95% CI)	*P*-value	HR(95% CI)	*P*-value	HR(95% CI)	*P*-value
BMI Traj							
OV-N	268 (50.4)	1.00 (referent)		1.00 (referent)		1.00 (referent)	
N-D	78 (69.7)	1.86(1.36–2.54)	<.0001	1.94(1.41–2.65)	<.0001	1.66(1.20–2.29)	0.003
OV-S	171 (48.9)	1.13(0.84–1.51)	0.41	1.19(0.88–1.60)	0.25	1.25(0.92–1.67)	0.15
OB-S	43 (69.2)	1.97(1.26–3.07)	0.003	2.01(1.28–3.14)	0.002	1.98(1.28–3.16)	0.003

NB: *BMI* body mass index, *NPHS* the National Population Health Survey, *N-D* normal weight-down, *OV-N* overweight-normal weight, *OV-S* overweight-stable, and *OB-S* obese-stable, *CI* confidence interval, *HR* hazard ratio

^a^Adjusted for age at baseline, race/ethnicity, education

^b^Adjusted for age at baseline, race/ethnicity, education, place of residence, disability, the probability of being physically active, smoking, and drinking, as well as the change patterns of the number of chronic conditions

Figure 2 illustrates that in women the GMP of the N-D group was 31.6%, and it started with a normal weight status at the age of 65 years, with an average BMI of 22.2, and slowly declined to an average BMI of 19.7 at age 96 years. Most women remained underweight or normal weight between the ages of 65 and 96 years. The GMP of the OV-N group was 41.9%. It started with an overweight status at age 65 years with an average BMI of 25.9. The average BMI gradually decreased to a normal weight status around 70–80 years of age then continued to decrease with increasing age. The average BMI did not reach underweight status, and the average BMI at age 96 years was 22.6. The GMP of the OB I-D group was 22.3%, the trajectory started with an obese class I status at age 65 years, with an average BMI of 30.2, and slowly declined to an average BMI of 25.9 at age 96 years. The GMP of the OB II-D group was 4.2% in women; it started with an obese class II status at age 65 years, with an average BMI of 37.6, and decreased to an average BMI of 34.6 at age 96 years.

Table 2 shows that 32.9%, 39.8%, 40.2%, and 36.7% of women died in the OV-N, N-D, OB I-D, and OB II-D groups, respectively. The N-D group was associated with a 32% (*P* = 0.03) increase in mortality risk when compared to the OV-N group, without controlling for other covariates. After adjusting for age, race/ethnicity and educational attainment, the N-D group was significantly associated with 31% more risk of mortality (*P* = 0.037). The other two groups (OB I-D and OB II-D) were not significantly different from the OV-N group in mortality risk in either the unadjusted or partly adjusted models. The interaction terms between the BMI trajectory and the number of chronic health conditions trajectory groups (*P* = 0.001) and the interaction term between age and physically active trajectory groups (*P* = 0.001) were both found to be significant (data available upon request) and the interaction term was included in the corresponding adjusted models. Following adjustment for all the covariates, the OB I-D group was significantly associated with a 61% (*P* < 0.001) increase in mortality risk among women who were assigned to the trajectory characterized with more than three chronic conditions. By contrast, the OB I-D group was associated with a 44% (*P* < 0.001) decrease in mortality risk among women who were assigned to the trajectory with less than three chronic conditions. Additionally, the proportional hazard assumption was examined and no significant violation of the assumption was found.

**Table 2:** Adjusted hazard ratios of BMI trajectories among women from cox proportional hazard models in the NPHS, 1994–2011

	Women						
	Unadjusted model		Adjusted demographics factorsc		Fully adjusted modeld		
						>3 conditions	<3 conditions
	N(%) death	HR(95% CI)	*P*-value	HR(95% CI)	P-value	HR(95% CI)	HR(95% CI)
BMI Traj							
OV-N	385 (32.9)	1.00 (ref.)		1.00(ref.)		1.00(ref.)	1.00(ref.)
N-D	291 (39.8)	1.32(1.03–1.69)	0.03	1.31(1.02–1.69)	0.04	1.23(0.86–1.77)	1.21(0.84–1.74)
OB I-D	205 (40.2)	1.25(0.94–1.65)	0.12	1.09(0.82–1.45)	0.54	1.61(1.12–2.31)	0.56(0.35–0.90)
OB II-D	39 (36.7)	1.05(0.55–1.98)	0.89	0.89(0.47–1.68)	0.71	0.71(0.30–1.67)	1.34(0.50–3.58)

NB: *BMI* body mass index, *NPHS* the National Population Health Survey, *N-D* normal weight-down, *OV-N* overweight-normal weight, *OB I-D* = obese I-down, and *OB II-D* obese II- down; *CI* confidence interval, *HR* hazard ratio

^c^Adjusted for age at baseline, race/ethnicity, education

^d^Fully adjusted for age at baseline, race/ethnicity, education, place of residence, disability, the probability of being physically active, smoking, and drinking, as well as the change patterns of the number of chronic conditions, and two interactions (the interaction between the BMI trajectory and the developmental of the number of chronic conditions trajectory and the interaction between age at baseline and the probability of being physically active trajectory)

## Discussion

This study investigates heterogeneity in body weight changes of seniors. Using latent class growth modelling, we identified four distinct BMI trajectory groups in elderly women and men. Overall, we observed that there’s no increase trend for all the BMI trajectories for both men and women, showing weight decrease for Canadian seniors in later years. The largest group, which consists of subjects with overweight status at age 65 then became normal weight in their 80s for both men and women. Our findings are well in line with previous BMI trajectories analysis for seniors aged 65 to 105 based on a community sample [32] and seniors aged 51 to 77 [13], but uniquely contributed to the age group 65 to 96 using a representative sample of Canadian seniors. Moreover, previous studies have reported that body weight increases up to the age of 70 years, then it either stabilize or decrease [33, 34].

The results of this investigation suggest that men who were overweight at age 65 and lost weight over time but never reached underweight status over time had the lowest mortality risk. Men who were obese at age 65 and remained obese over time had the highest mortality risk. Our findings suggest that if studies examine the associations between baseline BMI and mortality, then could have reported misleading conclusions that being overweight is associated with the lowest mortality risk [6]. Aiming for becoming/being normal weight is still necessary since it can potentially decrease mortality risk in elderly men after 65 years old, regardless of their current weight status. Zheng et al. found that individuals in the overweight-stable group had the lowest mortality risk followed by those in the overweight-obesity group, the normal weight-upward group, the class I obese-upward group, the normal weight-downward group, and the class II/III obese-upward groups [13]. The discrepancies may be come from the different age groups analyzed between the two studies. Additionally, the findings among men support the well-documented U-shaped association between BMI and mortality [12, 13, 35].

Interestingly, for women, the relationship between BMI trajectories and mortality risk is more complicated compared with men. We didn’t find significant mortality risk differences between the BMI trajectories after adjusting for all the confounders; the only exception is that women who were obese I at age 65 and with decreased body weight over time was associated with increased mortality risk for women with more than three chronic conditions. By contrast, in women who had less than three chronic conditions, being obese I at age 65 with decreased body weight over time was associated with decreased mortality risk. This evidence may be because for women who had more than three chronic conditions experienced disease-related weight loss, differing the beneficial impact on losing body weight for healthier women with less than three chronic conditions.

One of the most important strengths of this study is the usage of LCGM, which are able to examine the long-term obesity, lifestyles (physical activity, smoking and drinking), and chronic health conditions and their heterogeneities among the population. On the other hand, most studies generally use the baseline interview measurement to determine individuals’ lifestyle factors [13]. This study incorporates all available information reported in the 1994–2011 NPHS surveys, making the most use of the longitudinal data on physical activity, drinking, smoking and chronic conditions. The different findings between men and women also support the gender-related disparities in mortality risk found in previous research [15]. One limitation is that BMI and the chronic health conditions were self-reported. Although a more accurate measure of excess body fat would be ideal, BMI is the only indicator of body weight in the longitudinal NPHS data and no nationwide longitudinal data on measured body fat are available. In addition, no genetic data are collected in the longitudinal survey of NPHS.

## Conclusion

This study highlights the variations in BMI trajectories even for elderly population from the age of 65 onward. Distinct BMI trajectories and its associated mortality risk was found between Canadian men and women. Men with long-term obesity had the highest risk of mortality. On the other hand, men who were overweight at age 65 years and lost weight over time but never reached underweight over time had the lowest risk of mortality. Interestingly, obese women with decreasing BMI have different mortality risks, depending on their chronic health conditions. Our findings indicate that for men, aiming for becoming/being normal weight regardless of their current weight status can potentially decrease mortality risk; while this evidence may be not sure for women. On the other hand, for women, disease management seems a more efficient way to decrease mortality risk rather than focusing on body weight alone. The findings of this study provide new insights into the debate concerning the associations between BMI and mortality risk among seniors.

## Figures and Tables

**Fig. 3:**
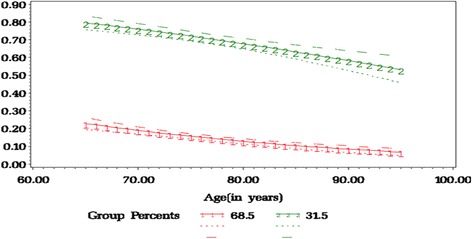
The probability of being physically active trajectories for seniors (65–96 years), with 95% confidence intervals (four group model, no covariates included), NPHS, 1994–2011

**Fig. 4:**
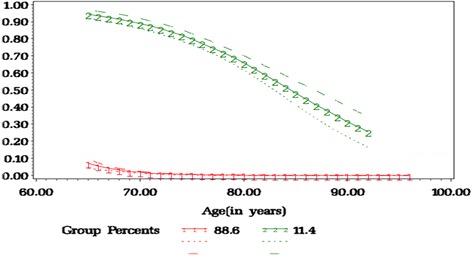
The probability of smoking trajectories for seniors (65–96 years), with 95% confidence intervals (four group model, no covariates included), NPHS, 1994–2011

**Fig. 5:**
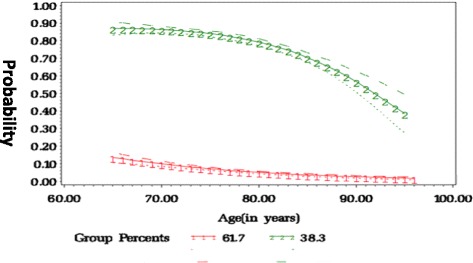
The probability of drinking trajectories for seniors for seniors (65–96 years), with 95% confidence intervals (four group model, no covariates included), NPHS, 1994–2011

**Fig. 6:**
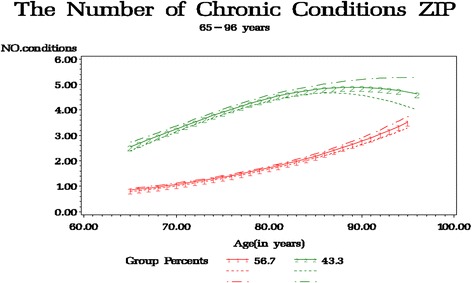
The trajectories of the number of chronic conditions for seniors (65–96 years), with 95% confidence intervals (four group model, no covariates included), NPHS, 1994–2011

## Appendix

**Table 3:** Results of the multivariable proportional hazards Cox regression model adjusted for age at baseline, race/ethnicity, educational level, place of residence, disability, the probability of being physically active, smoking, drinking, and the development of the number of chronic conditions for women (fully adjusted model)

Analysis of maximum likelihood estimates					
Covariates	Parameter estimate	Standard error	Pr > ChiSq	Hazard ratio	95% HR Conf. limit
BMI Traj					
N-D	0.19	0.19	0.32		
OB I-D	−0.59	0.24	0.02		
OB II-D	0.30	0.50	0.55		
OV-D(ref.)					
The number of chronic conditions					
More than 3	−0.07	0.19	0.70		
Less than 3					
BMI Traj* the number of chronic conditions			0.001		
N-D more than 3	0.02	0.26	0.928		
OB I-D more than 3	1.06	0.30	0.0005		
OB II-D more than 3	−0.64	0.66	0.336		
OV-D (ref.)					
Age					
	0.12	0.02	<.0001		
PA					
Active	8.01	2.54	0.002		
Inactive(ref.)					
Age * PA			0.001		
Active	−0.12	0.04	0.001		
Inactive(ref.)					
Race					
Non-white	−0.43	0.34	0.20	0.65	0.34–1.26
White (ref.)					
Disability					
Yes	0.48	0.13	0.0002	1.62	1.26–2.10
No (ref.)					
Education (if high school graduate)					
Yes	−0.23	0.12	0.05	0.79	0.63–0.99
No(ref.)					
Place of residence					
Rural	−0.30	0.12	0.01	0.76	0.59–0.97
Urban(ref.)					
Smoking					
smoker	0.98	0.16	<0.0001	2.57	1.86–3.56
Non-smoker (ref.)					
Drinking					
Regular drinker	−0.11	0.13	0.40	0.90	0.70–1.15
Non-drinker(ref.)					

**Table 4:** Results of the multivariable proportional hazards Cox regression model adjusted for age at baseline, place of residence, disability, the probability of being physically active, smoking, drinking, and the development of the number of chronic conditions for men (fully adjusted model)

Analysis of maximum likelihood estimates					
Covariates	Parameter estimate	Standard error	Pr > ChiSq	Hazard ratio	95% HR Conf. limit
BMI Traj					
N-D	0.51	0.17	0.002	1.66	1.20–2.30
OV-S	0.22	0.15	0.15	1.25	0.92–1.68
OB -S	0.68	0.23	0.003	1.98	1.26–3.13
OV-D(ref.)					
The number of chronic conditions					
More than 3	0.37	0.14	0.007	1.45	1.11–1.90
Less than 3(ref.)					
Age					
	0.11	0.02	<.0001	1.11	1.08–1.15
Race					
Non-white	0.12	0.32	0.70	1.13	0.60–2.13
White (ref.)					
Education (if high school graduate)					
Yes	−0.05	0.13	0.70	0.95	0.73–1.23
No(ref.)					
PA					
Active	−0.57	0.14	<.0001	0.60	0.43–0.74
Inactive(ref.)					
Disability					
Yes	0.34	0.14	0.01	1.41	1.08–1.84
No (ref.)					
Place of residence					
Urban	−0.24	0.13	0.06	0.79	0.61–1.01
Rural(ref.)					
Smoking					
Smoker	1.06	0.16	<.0001	2.89	2.11–3.98
Non-smoker (ref.)					
Drinking					
Regular drinker	−0.17	0.12	0.17	0.84	0.66–1.07
Non-drinker(ref.)					

## Abbreviations

AvePP: Average posterior probability of group membership
BMI: Body mass index
CGM: Conventional growth modelling
GMP: Group membership probability
HR: Hazard ratio
LCGM: Latent class growth modelling
NPHS: National Population Health Survey
PA: Physical activity
